# Herpes Simplex Virus Type 2, Genital Ulcers and HIV-1 Disease Progression in Postpartum Women

**DOI:** 10.1371/journal.pone.0019947

**Published:** 2011-05-26

**Authors:** Alison C. Roxby, Alison L. Drake, Grace John-Stewart, Elizabeth R. Brown, Daniel Matemo, Phelgona A. Otieno, Carey Farquhar

**Affiliations:** 1 Department of Medicine, University of Washington, Seattle, Washington, United States of America; 2 Department of Global Health, University of Washington, Seattle, Washington, United States of America; 3 Department of Epidemiology, University of Washington, Seattle, Washington, United States of America; 4 Department of Biostatistics, University of Washington, Seattle, Washington, United States of America; 5 Department of Obstetrics and Gynaecology, University of Nairobi, Nairobi, Kenya; 6 Centre for Clinical Research, Kenya Medical Research Institute, Nairobi, Kenya; Indiana University, United States of America

## Abstract

**Background:**

Co-infection with herpes simplex virus type 2 (HSV-2) has been associated with increased HIV-1 RNA levels and immune activation, two predictors of HIV-1 progression. The impact of HSV-2 on clinical outcomes among HIV-1 infected pregnant women is unclear.

**Methods:**

HIV-1 infected pregnant women in Nairobi were enrolled antenatally and HSV-2 serology was obtained. HIV-1 RNA and CD4 count were serially measured for 12–24 months postpartum. Survival analysis using endpoints of death, opportunistic infection (OI), and CD4<200 cells µL, and linear mixed models estimating rate of change of HIV-1 RNA and CD4, were used to determine associations between HSV-2 serostatus and HIV-1 progression.

**Results:**

Among 296 women, 254 (86%) were HSV-2-seropositive. Only 30 (10%) women had prior or current genital ulcer disease (GUD); median baseline CD4 count was 422 cells µL. Adjusting for baseline CD4, women with GUD were significantly more likely to have incident OIs (adjusted hazard ratio (aHR) 2.79, 95% CI: 1.33–5.85), and there was a trend for association between HSV-2-seropositivity and incident OIs (aHR 3.83, 95% CI: 0.93–15.83). Rate of change in CD4 count and HIV-1 RNA did not differ by HSV-2 status or GUD, despite a trend toward higher baseline HIV-1 RNA in HSV-2-seropositive women (4.73 log10 copies/ml vs. 4.47 log10 copies/ml, P = 0.07).

**Conclusions:**

HSV-2 was highly prevalent and pregnant HIV-1 infected women with GUD were significantly more likely to have incident OIs than women without GUD, suggesting that clinically evident HSV-2 is a more important predictor of HIV-1 disease progression than asymptomatic HSV-2.

## Introduction

HIV-1 and herpes simplex virus type 2 (HSV-2) co-infection presents important implications during pregnancy for women and their children. Prior studies in HIV-1 infected pregnant women have demonstrated associations between HSV-2 and genital ulcers and perinatal HIV-1 transmission [1], [2], [3], [4], and co-infected women have increased genital shedding of HIV-1 [5]. Women with HIV-1 in developing countries have increased morbidity in pregnancy and postpartum [6], and more perinatal complications than HIV-1-seronegative women [7], [8]. Addressing the implications of HSV-2 infection in pregnancy and postpartum may be a tool to improve the health of HIV-1 infected mothers and their infants.

HSV-2 co-infection has been associated with increased heterosexual acquisition [9] and transmission [10] of HIV-1, increased levels of cellular immune activation [11], and elevated HIV-1 viral loads [12], [13]. Antivirals targeting HSV-2 are low cost, safe, and well-tolerated, making them attractive novel therapeutic tools. Multiple short randomized trials of acyclovir or valacyclovir in co-infected patients have shown 0.25 to 0.5 log_10_ copies/ml reductions in HIV-1 viral load [10], [14], [15], [16], [17], [18], [19]. A multisite randomized controlled trial of daily acyclovir in over 3,000 HIV-1-seropositive, HSV-2-seropositive African adults demonstrated that HSV-2 suppression not only produced a sustained reduction in HIV-1 viral load over a 2-year period in participants on acyclovir [10], but also reduced risk of HIV-1 disease progression events by 16% when compared to placebo [20]. Suppression of HSV-2 with acyclovir or valacyclovir has been hypothesized as a strategy to mitigate the consequences of chronic HIV-1 infection in sub-Saharan Africa, where more than 80% of people with HIV-1 are HSV-2-seropositive. However, HIV-1 infected pregnant women have been excluded from trials of HSV-2 suppression.

To evaluate associations between HSV-2 and HIV-1 disease in pregnant and postpartum women, we conducted a retrospective cohort study to determine the effect of HSV-2 serostatus and GUD on HIV-1 disease progression in HIV-1 infected pregnant women followed for 12 to 24 months after delivery.

## Methods

### Study setting and population

HIV-1-seropositive pregnant women in Nairobi, Kenya were recruited from Nairobi City Council clinics and enrolled into a prospective cohort study of immunological markers, morbidity and infant feeding practices, as described previously [3]. The first 216 women, enrolled between 1999 and 2002, were followed for 12 months; the remaining 319 women, enrolled between 2002 and 2005, were followed for 24 months.

### Ethics

Written informed consent was obtained from all study subjects in the cohort. Human experimentation guidelines from the US Department of Health and Human Services were followed. Ethical approvals were obtained from the institutional review board of the University of Washington and the ethics review committee of Kenyatta National Hospital (KNH) and the Kenya Medical Research Institute (KEMRI).

### Clinical procedures

Women were followed during pregnancy and postpartum with regular physical exams, and plasma samples were taken at postpartum months 1, 3, 6, 9, and 12, and then quarterly for those followed during the second postpartum year. At enrollment, a pelvic exam was done and the presence of any ulcer was recorded. Women were classified as having genital ulcer disease (GUD) if they had either reported having a history of an ulcer prior to enrollment or had an ulcer observed during the exam at enrollment. All women were screened for syphilis, the other main cause of GUD in this population, with baseline rapid plasma reagin (RPR) testing, and treated if positive. The following opportunistic infections (OIs) were recorded: incident pulmonary or extrapulmonary tuberculosis, herpes zoster, *Pneumocystis jirovecii* pneumonia (PCP), Kaposi's sarcoma (KS), meningitis or encephalitis. These diagnoses were made in study clinic or were abstracted from records of hospitalized participants.

Women were provided zidovudine (ZDV) prophylaxis according to standard prevention of mother-to-child transmission (PMTCT) protocols in Kenya at that time, which included short courses of ZDV beginning at 34–36 weeks gestation through delivery. Women with CD4 counts≤200 cells/µl received co-trimoxazole prophylaxis per Kenyan guidelines during the study period and were referred for highly-active antiretroviral therapy (HAART), although access to HAART in Kenya remained limited until 2003. Consistent with clinical practice in Kenya at the time, acyclovir was not used for treatment of GUD, but some women received it for severe herpes zoster.

### Laboratory methods

Cryopreserved plasma samples from the date of study enrollment were identified from women in the cohort. HSV-2 antibody was detected using HSV-2 enzyme-linked immunosorbency assays (ELISA) (HerpeSelect, Focus Diagnostics, Cypress, CA, USA), performed at University of Washington in 2007 (first 175 specimens) and University of Nairobi in 2009 (remaining 124 specimens). A cutoff index value ≥3.5 was used to determine a positive result. In Seattle, positive and equivocal results were repeated with Western blot testing and participants were considered positive if Western blot testing was positive. In Nairobi, Western blot testing was not done but samples with indeterminate results were re-tested; if a result remained indeterminate, the participant was excluded from analysis. CD4 count was measured in Nairobi with a FACScan flow cytometer (BD Biosciences, San Jose, CA USA), with semiannual proficiency testing performed. Plasma HIV-1 RNA levels were quantified at the Fred Hutchinson Cancer Research Center in Seattle using a transcription-mediated amplification assay (Gen-Probe, San Diego, CA USA), which has been validated to quantify prevalent HIV-1 subtypes in Kenya [21]. Maternal plasma was tested for syphilis at enrollment using the rapid plasma reagin (RPR) (Becton Dickinson, Cockeysville, MD) and confirmed using the *Treponema pallidum* hemagglutination assay (Randox Laboratories Ltd, Ardmore, Crumlin, UK).

### Statistical methods

To determine baseline correlates of HSV-2-seropositivity in the cohort, univariate analyses were performed using Chi-squared, Fisher's exact test, and Student t-tests. Maternal HIV-1 disease progression was defined using three separate endpoints: death, CD4≤200 cells/µl, and first OI. Participants were censored after the first occurrence of an event. The following combined outcomes were also evaluated: 1) death or CD4≤200 cells/µl, 2) death or OI, and 3) death, CD4≤200 cells/µl or OI. Cumulative incidence was estimated using the Kaplan-Meier curves. Associations between disease progression and baseline risk factors were assessed using Cox proportional hazards regression, adjusted for baseline CD4 count. Data were also censored if any of the following occurred: death, start of HAART, or second pregnancy.

To estimate rates of change of postpartum CD4 count and HIV-1 RNA levels, linear mixed effects models with random slopes were constructed. HIV-1 RNA levels and CD4 counts during pregnancy were excluded from linear mixed effects models. A locally weighted scatterplot smoother was applied to scatterplots of CD4 and plasma HIV-1 RNA level. Stata Version 10.0 (College Station, Texas USA) software was used for statistical analysis.

## Results

### Study population

Of the 535 women in the original cohort, 299 women had baseline plasma samples available for HSV-2 antibody assays. HSV-2 serostatus was ascertained for 296 women; their characteristics are summarized in Table 1. Baseline characteristics of women with HSV-2 serology results were similar to the characteristics of all women in the original cohort (data not shown) [22]. The median age of women was 25 years and 95% were married or partnered. Of those with HSV-2 serology results, 254 (86%) were HSV-2-seropositive and 30 (10%) had prior or current GUD. Nineteen (6%) women reported GUD prior to enrollment and all were also HSV-2-seropositive. Ten women (3%) were diagnosed with syphilis at baseline; only one woman with serologic syphilis had an observed or reported ulcer; verifying that most genital ulcers in our cohort were due to HSV-2. Median baseline CD4 count was 422 cells/µl and 12% of women had CD4≤200 cells/µl.

**Table 1:** Characteristics of HIV-1 Infected Women at Enrollment, and by HSV-2 Antibody Status or GUD.

	Median value (IQR) Or (N) %	Mean or N (%)	Mean or N (%)	*P*-value	Mean or N (%)	Mean or N (%)	*P*-value
Characteristic	All women N = 296	HSV-2-seropositive N = 254	HSV-2-seronegative N = 42		Ever GUD N = 30	Never GUD N = 266	
Age (years)	25 (22–28)	25.5	25.2	0.77	26.2	25.3	0.37
Parity*	1 (1–2)	1.5	1.4	0.64	1.7	1.5	0.52
Lifetime number of sex partners	3 (2–4)	3.4	2.5	0.13	3.1	3.3	0.80
History of STI	39 (13%)	34 (13%)	5 (12%)	0.79	5 (17%)	34 (13%)	0.55
Syphilis at enrollment**	10 (3%)	10 (4%)	0 (0)	0.21	1 (3%)	9 (3%)	0.73
History of GUD	19 (6%)	19 (8%)	0 (0)	0.05	-	-	-
GUD on exam	13 (4%)	12 (5%)	0 (0)	0.13	-	-	-
Ever GUD	30 (10%)	30 (12%)	0 (0)	0.01	-	-	-
HSV-2-seropositive	254 (86%)	-	-	-	30 (100%)	0 (0%)	0.02
Baseline CD4 (cells/µl)#	422 (288–617)	465	467	0.96	458	466	0.86
CD4≤200 cells/µl#	35 (12%)	33 (13%)	2 (5%)	0.14	4 (13%)	31 (12%)	0.83
Baseline log_10_ HIV-1 RNA level#	4.75 (4.22–5.31)	4.73	4.46	0.07	4.82	4.68	0.40

Methods: Chi-squared or Fisher's exact test for dichotomous variables and t-test for continuous variables.

GUD = genital ulcer disease. IQR = interquartile range. STI = sexually transmitted infection.

*N = 293,

**N = 294,

# N = 288.

Mean follow-up was 18 months, during which time 19 deaths occurred; 17 among HSV-2-seropositive women (hazard ratio [HR] 1.33, 95% confidence interval [CI]: 0.32–6.05). OIs were identified in 45 (15%) women, including 28 with tuberculosis, 16 with herpes zoster, 4 with meningitis, 2 with PCP and one with KS. Six (2%) women had more than one OI during follow-up; only the first OI was used for analysis.

### Correlates of HSV-2 seropositivity and genital ulcers

Only HSV-2-seropositive women reported a history of GUD (8% vs. 0% of HSV-2-seronegative women, *P* = 0.05) and they tended to be more likely to have a CD4 count≤200 cells/µl at baseline compared to HSV-2-seronegative women (13% vs. 5%, *P* = 0.14). There was also a trend for higher mean baseline HIV-1 log_10_ viral loads among HSV-2-seropositive women (4.73 log_10_ copies/ml vs. 4.46 log_10_ copies/ml, *P* = 0.07). Reported history of sexually transmitted infection (STI) was similar in both groups (12% vs. 13%, *P* = 0.79), and there was no difference in age, parity, or number of lifetime sexual partners between HSV-2-seropositive and seronegative women (*P*>0.05 for all) (Table 1). All 30 women in the study with GUD were also HSV-2-seropositive and had similar STI history, syphilis prevalence, baseline CD4 counts and HIV-1 log_10_ viral loads when compared to women without GUD (Table 1). Of those with GUD, 13 women (5%) had an ulcer seen at the baseline exam.

### HSV-2-seropositivity, GUD, and HIV-1 disease progression

**Figure 1:**
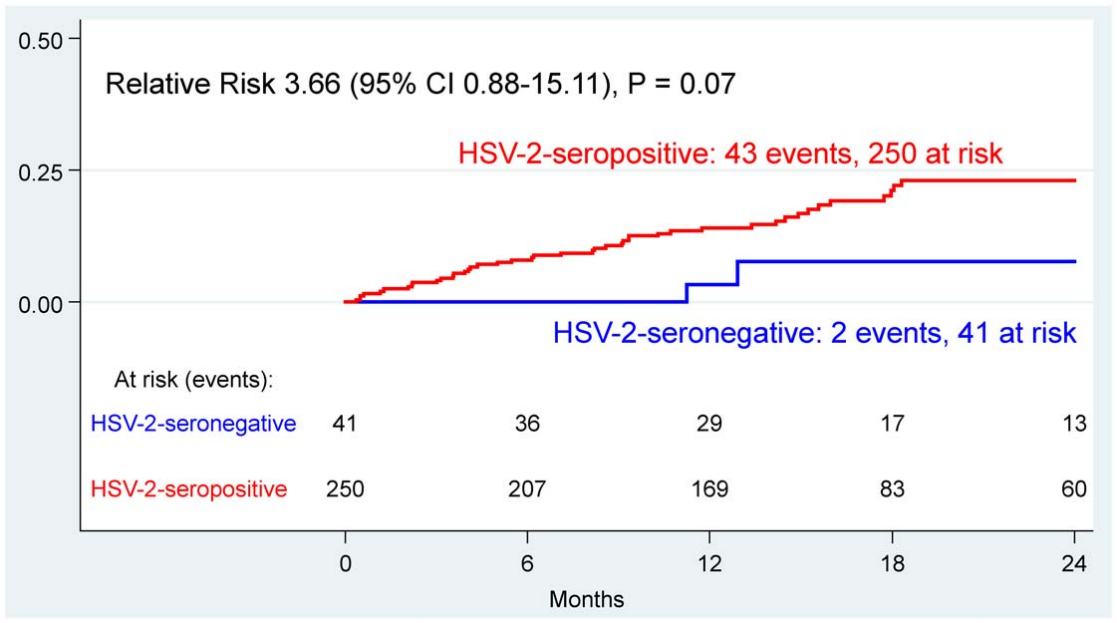
Risk of Opportunistic Infection, by HSV-2 Serostatus (Kaplan-Meier Estimates). Opportunistic infection is defined as: first episode of herpes zoster, *Pneumocystis jirovecii* pneumonia, tuberculosis, Kaposi sarcoma, meningitis, or encephalitis. Relative risk calculated using Cox regression. Time = months since delivery.

**Figure 2:**
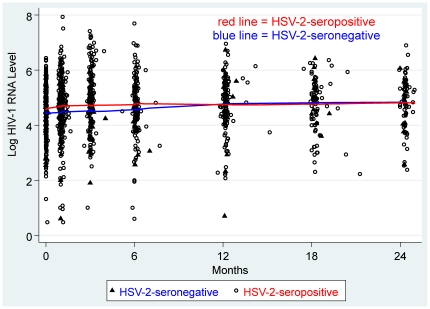
Scatterplot of Log_10_ HIV-1 RNA Level Over Time, by HSV-2 Serostatus at Baseline. Points represent individual measures of log_10_ HIV-1 RNA levels. Curves represent locally weighted smoothed curves of log_10_ HIV-1 RNA levels over time, by HSV-2 serostatus. Time = months since delivery.

There was a trend toward an association between HSV-2-seropositivity and incident OI; 17% of the HSV-2-seropositive women had an incident OI during follow-up, compared to 5% of the HSV-2-seronegative women (HR 3.66, 95% CI: 0.88–15.11, *P* = 0.07) (Figure 1). In a model adjusted for baseline CD4, the relationship between HSV-2-seropositivity and OI persisted (Adjusted HR (aHR) 3.83, 95% CI: 0.93–15.83, *P* = 0.06) (Table 2). Progression to CD4≤200 cells/µl was similar between HSV-2-seropositive and seronegative women (HR 1.15, 95% CI: 0.52–2.53, *P* = 0.74), and changes in plasma HIV-1 RNA did not differ significantly by HSV-2 serostatus (Figure 2). HSV-2 serostatus did not show a significant effect on rates of change in CD4; the average rate of change of CD4 cells/µl/month was −3.42 cells/µl/month in the HSV-2-seronegative group and −4.22 cells/µl/month in the HSV-2-seropositive group (*P* = 0.57).

**Table 2:** Risk of Postpartum HIV-1 Progression Event by HSV-2 Serostatus and GUD.

Event			Total events (%)	aHR*	95% CI	*P*-value
	**HSV-2-seropositive N = 254**	**HSV-2- seronegative N = 42**				
Death	17 (7%)	2 (5%)	19 (6%)	1.39	0.32–6.05	0.66
CD4≤200 cells/µl	50 (20%)	7 (17%)	57 (19%)	1.16	0.52–2.56	0.72
Opportunistic Infection	43 (17%)	2 (5%)	45 (15%)	3.83	0.93–15.83	0.06
	**Any GUD N = 30**	**No GUD N = 266**				
Death	3 (10%)	16 (6%)	19 (6%)	1.17	0.33–4.08	0.80
CD4≤200 cells/µl	6 (20%)	51 (19%)	57 (19%)	0.63	0.26–1.49	0.29
Opportunistic Infection	9 (30%)	36 (14%)	45 (15%)	2.79	1.33–5.85	0.007

GUD = genital ulcer disease; CI = Confidence interval; aHR = Adjusted hazard ratio.

*adjusted for Baseline CD4 count.

Opportunistic infection is defined as: herpes zoster, *Pneumocystis jirovecii* pneumonia, tuberculosis, Kaposi sarcoma, meningitis, encephalitis.

**Figure 3:**
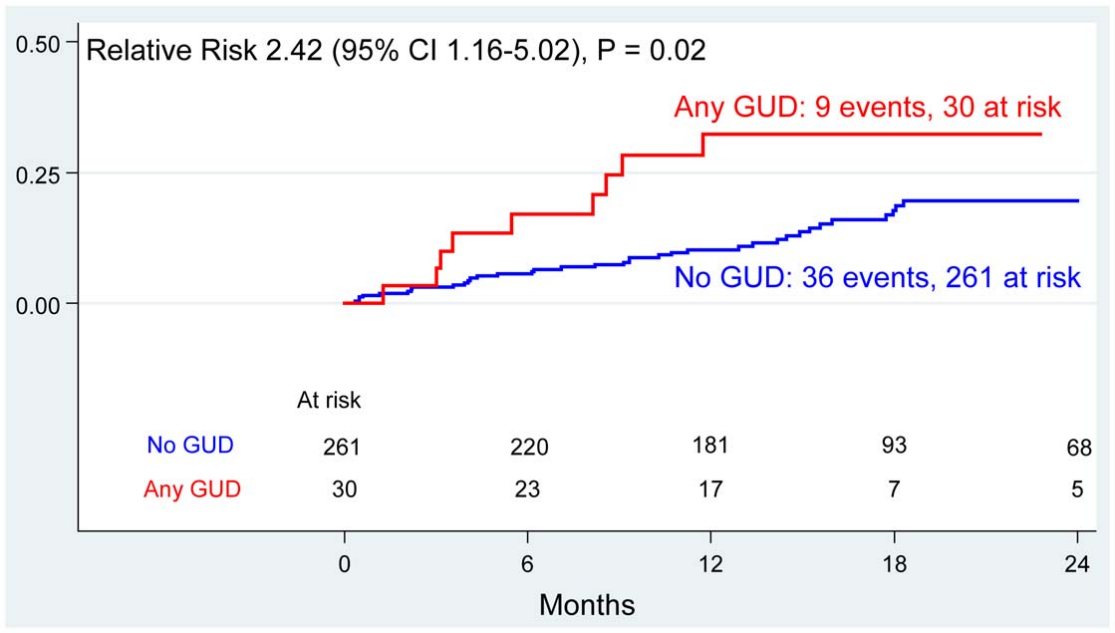
Risk of Opportunistic Infection, by GUD (Kaplan-Meier Estimates). Opportunistic infection is defined as: first episode of herpes zoster, *Pneumocystis jirovecii* pneumonia, tuberculosis, Kaposi sarcoma, meningitis, or encephalitis. Relative risk calculated using Cox regression. Time = months since delivery.

GUD was found to be significantly associated with incident OIs (HR 2.42, 95% CI: 1.16–5.02, *P* = 0.02) (Figure 3), and this relationship strengthened after adjusting for baseline CD4 (aHR 2.79, 95% CI: 1.33–5.85, *P* = 0.007) (Table 2). Nearly one-third (30%) of the women with GUD had an OI during follow-up, compared with only 14% of the women who had not experienced GUD. The rate of change of HIV-1 RNA was similar for women with and without GUD. The rate of decline of CD4 count was −4.34 cells/µl/month for those without GUD vs. −2.34 cells/µl/month for those with GUD, but this difference was not statistically significant (*P* = 0.25).

## Discussion

In this cohort of 296 pregnant and postpartum women in Kenya, we evaluated HIV-1 disease progression in HSV-2 co-infected participants. Women with GUD were more likely to experience an OI during the follow-up period, and this relationship persisted after controlling for CD4 count at study entry. HSV-2-seropositive women also exhibited this trend toward more OIs.

HSV-2-seropositive women with genital ulcers may differ from co-infected women with asymptomatic HSV-2; and this difference cannot be explained by CD4 count alone. It is possible that symptomatic genital ulcers may identify co-infected persons with different immunologic characteristics from those who are seropositive for HSV-2 but do not develop or recognize GUD. Our study is not the first to observe the importance of GUD as a possible marker of HIV-1 disease progression. In both incident and prevalent HIV-1 infection, patients in Rakai, Uganda with GUD were noted to have higher plasma viral loads than those who were HSV-2-seropositive without GUD, an effect which persisted after stratifying by disease stage [23]. A study of a cohort of 1,938 HIV-1/HSV-2 co-infected women in the Women's Interagency Health Study (WIHS) noted that women with GUD had modest increases in plasma HIV-1 viral load over time, as well as lower CD4 counts, when compared with asymptomatic women, even after adjusting for baseline CD4 counts [24]. Chronic antiviral treatment for GUD has been shown to increase long term survival of HIV-1 infected patients in several randomized trials [25], [26], [27] and one meta-analysis [28].

While acyclovir therapy has been shown to have clinical and virological benefits for co-infected HIV-1-seropositive adults (both with and without symptomatic disease), it has not been studied during pregnancy and postpartum. Our analysis was motivated by the hypothesis that demonstrating increased HIV-1 disease progression in co-infected women during pregnancy and postpartum could provide a rationale for HSV-2 antiviral therapies as an adjunct treatment for HIV-1. Our results suggest that women with GUD are a subgroup that may benefit from anti-herpes treatment or prophylaxis. Furthermore, using GUD to determine eligibility for implementation of anti-herpes treatment would eliminate the barrier of diagnostic testing for HSV-2 seropositivity. Lingappa *et al* showed a 16% reduction in HIV-1 disease progression for HIV-1/HSV-2 co-infected adults when using acyclovir for herpes suppression. It is also possible that during pregnancy and postpartum, anti-HSV-2 therapy may provide additional suppression of HIV-1 viral load, and could add to interventions to prevent transmission of HIV-1 to infants.

Our study had several strengths and limitations. Women in this pre-HAART cohort were closely monitored for illness and laboratory markers of disease progression, experienced high rates of morbidity and mortality [6], and there was minimal loss to follow-up. Low numbers of HSV-2-seronegative women, however, limited our power to detect associations between HSV-2 seropositivity and dichotomous outcomes such as mortality or CD4 count≤200 cells/µl. Using the risk estimate of HR = 0.84 reported by Lingappa *et al* [20] for the effect of acyclovir on similar dichotomous outcomes, a sample size of at least 2,000 participants would be required to show a similar result. Thus, our sample lacked statistical power to definitively exclude an association between HSV-2 serostatus and HIV-1 disease progression in postpartum women. However, by evaluating change in quantitative measures of CD4 and HIV-1 RNA over time, power was enhanced.

Limitations in our analysis include the challenges of identifying prior and current GUD, due to symptoms of varying severity. Women may not have recalled prior GUD at study entry. Incident ulcers were not systematically assessed, therefore we were unable to include the impact of incident ulcers in our analysis. Also, there was possible miscategorization of HSV-2-seronegative women who were at ongoing risk of acquiring HSV-2 infection and may have seroconverted during follow-up.

In summary, this study demonstrates an increased incidence of OIs among pregnant and postpartum women with HSV-2 infection and GUD. A randomized clinical trial in Kenya of HSV-2 suppression in pregnant and postpartum women with HIV-1 and HSV-2 co-infection was recently concluded; this trial may provide further information on effects of herpes suppression on maternal HIV-1 disease progression in co-infected pregnant and postpartum women. Given the high mortality rates and poor health outcomes among HIV-infected women in sub-Saharan Africa and the high rates of HIV/AIDS-related orphans when mothers die prematurely, herpes suppressive therapy should be considered as an additional tool to improve maternal health and survival among women with HIV-1 and GUD.
